# Therapeutic Effect of Epilepsy Diagnosis Using Video-Electroencephalography in an Adult Misdiagnosed With Apathy and Psychogenic Nonepileptic Seizures: A Case Report

**DOI:** 10.7759/cureus.77093

**Published:** 2025-01-07

**Authors:** Masahiro Umeda, Go Taniguchi, Hideo Kato, Chihiro Nakata, Eiji Nakagawa

**Affiliations:** 1 Department of Epileptology, National Center of Neurology and Psychiatry, Tokyo, JPN

**Keywords:** asd ( autism spectrum disorders), epilepsy case report, overinvolvement, psychiatric symptoms, psychogenic nonepileptic seizures (pnes)

## Abstract

Patients with epilepsy frequently present with comorbid psychiatric symptoms such as depression, anxiety, and apathy. In cases of drug-resistant epilepsy with prominent psychiatric symptoms, misdiagnosis as psychogenic nonepileptic seizures (PNES) is common. Video-electroencephalogram (VEEG) can play a critical role in differentiating PNES from epileptic seizures. VEEG has also been reported to have therapeutic benefits in PNES. However, its effects on psychiatric symptoms related to epilepsy, excluding PNES, have not been reported. Herein, we report a case of apathy that occurred after the onset of epilepsy and improved following a definitive diagnosis using VEEG.

A 25-year-old woman exhibiting traits consistent with autism spectrum disorder was referred for the differential diagnosis of PNES. Social activity decreased after the onset of seizures, and marked apathy was observed after the mother began over-involvement. The seizures initially presented as focal clonic seizures and evolved into focal to bilateral tonic-clonic seizures, but over time, seizures with impaired consciousness began to appear. Combined with the presence of severe apathy, all seizures were misdiagnosed as PNES. After epilepsy was confirmed by VEEG, the patient’s apathy improved markedly, and the frequency of seizures decreased. When apathy worsened again due to seizure recurrence after discharge, VEEG was ineffective. Following the initiation of occupational therapy (OT), apathy gradually improved, and no further exacerbation of apathy was observed, even in the presence of seizures.

This case underscores the therapeutic potential of VEEG. The patient was initially misdiagnosed with PNES due to the combination of severe apathy and seizures suspected to be PNES. However, the correct diagnosis and educational effect provided by VEEG contributed to an improvement in apathy. Following the diagnosis via VEEG, the frequency of seizures also decreased without any changes to antiepileptic medications, suggesting that the improvement in psychiatric symptoms positively impacted the control of epileptic seizures. Although VEEG was ineffective during the recurrence of apathy triggered by seizures, gradual improvement was observed through OT and encouragement of independence. Furthermore, continued OT in outpatient settings prevented further worsening of apathy related to seizures.

In this case, apathy improved following a definitive diagnosis of epilepsy using VEEG. VEEG not only facilitated accurate epilepsy diagnosis but also improved apathy and seizure control, underscoring both its diagnostic and therapeutic value. Further research is warranted to explore standardized assessments of psychiatric symptoms before and after VEEG to optimize care in complex epilepsy cases.

## Introduction

Epilepsy is conceptually defined as a brain disorder characterized by a persistent predisposition to epileptic seizures [1], including tonic-clonic, focal clonic, absence, and focal impaired awareness seizures. The annual incidence of epilepsy has been estimated at 61.4 per 100,000 people [2], making it one of the leading neurological diseases. Epilepsy remains a stigmatized condition, often resulting in discrimination and social isolation. Furthermore, the significant social limitations imposed by the disorder, coupled with the unpredictability of seizures, can contribute to diminished self-esteem and the development of depression [3]. Patients with epilepsy frequently present with comorbid psychiatric symptoms such as depression, anxiety, and apathy, which can significantly impair their quality of life [4]. Notably, individuals with epilepsy have a two-to-five times higher risk of developing psychiatric disorders, and one in three people with epilepsy will be diagnosed with a psychiatric disorder during their lifetime [3]. Pharmacological and psychological treatments are provided based on the psychiatric symptoms; however, these are often refractory to treatment [5].

Apathy, characterized by reduced motivation, interest, and emotional engagement, is observed in chronic neurological disorders such as Parkinson's disease, Alzheimer's disease, stroke, and epilepsy. It is also reported in various psychological conditions [6]. It can decrease patients' quality of life, increase their dependence on caregivers, and impair their participation in social, self-care, and rehabilitation activities, thereby heightening caregiving burdens and care-related needs [7].

Psychogenic nonepileptic seizures (PNES) are episodes of altered movement, sensation, or experience distinguished from epileptic seizures by the lack of associated ictal abnormal electrical brain discharges. About one-quarter of patients referred to specialist centers for apparent drug-resistant epilepsy are found to be misdiagnosed. PNES often develop based on underlying psychiatric conditions [8]. Consequently, in cases of drug-resistant epilepsy with prominent psychiatric symptoms, misdiagnosis as PNES is common. Therefore, video-electroencephalogram (VEEG), which enables simultaneous recording of video and electroencephalography, is crucial for distinguishing between epilepsy and PNES. Furthermore, VEEG has been reported to have therapeutic benefits in PNES [8]. However, to the best of our knowledge, its effect on psychiatric symptoms related to epilepsy, excluding PNES, has not been reported.

Herein, we report a case of apathy following the onset of epilepsy, which was initially misdiagnosed as PNES. Notably, the patient's apathy improved after a definitive diagnosis of epilepsy using VEEG. The patient also experienced decreased seizure frequency without any changes to antiepileptic medication.

## Case presentation

A 25-year-old woman was referred to our department of epileptology for the differential diagnosis of PNES. Three years before visiting our hospital, the patient began managing household tasks and caring for her mother due to her mother’s worsening anxiety. Despite graduating from college two years prior, the patient had not sought employment to care for her mother. One and a half years before visiting our hospital, the mother was hospitalized for obsessive-compulsive disorder (OCD). Two months before the admission, the patient began to experience monthly involuntary jerks in the hands and feet, as well as unknown onset tonic-clonic seizures lasting 1-2 minutes. There were no identifiable triggers for the seizures, and no changes were observed in consciousness, aura, or postictal symptoms. Four months after the seizures began, the patient was initially treated with lacosamide (LCM), an anti-seizure medication, at a dosage of 100 mg/day (50 mg twice daily). Six months later, the dose was increased to 200 mg/day (100 mg twice daily) for more than one year. However, the seizures persisted and gradually adopted a weekly frequency, leading to gradual withdrawal from social activities. The patient stopped socializing with friends, stopped going out for shopping, and started staying at home. Although the patient remained independent in her daily activities, she rarely spoke to her family anymore.

The mother was discharged after a one-year hospitalization; shortly thereafter, she began providing excessive daily assistance to the patient, which coincided with a decline in spontaneity and an increase in reticence. The patient required assistance with basic tasks such as eating and bathing and experienced episodes of loss of consciousness in specific situations, such as brushing teeth, bathing, and toileting, which lasted for several minutes daily. Additionally, seizures lasting approximately 30 minutes were observed on several occasions, though these occurred less frequently than once per month. A previous physician had diagnosed all episodes as PNES based on an increasing number of episodes of altered consciousness suspected to be PNES, as well as the noticeable decline in activities of daily living (ADL). The diagnosis of PNES was explained to the patient, and the family was instructed not to overreact to the seizures. However, psychotherapy was not prescribed because of the patient's mutism. After six months without improvement, the patient was referred to our hospital.

The patient had a history of non-febrile seizures. At age two, the patient experienced two brief tonic-clonic seizures. From the age of two to nine years, the patient was treated with valproic acid, after which she remained seizure-free, and follow-up was discontinued. Regarding family history, the patient’s 52-year-old mother was previously hospitalized four times, totaling 18 months, due to OCD and had been continuously taking medications, including selective serotonin reuptake inhibitors. Moreover, the patient’s history from childhood to the present had been characterized by limited interaction with anyone other than her mother, with communication primarily occurring through text messages. Additionally, the patient demonstrated notable preferences, for example, wearing winter socks even during summer. The patient was not evaluated for autism spectrum disorder (ASD) during childhood. However, based on difficulties in social communication, as well as restricted and repetitive behaviors, ASD traits were considered to be present. Before visiting our department, the patient had never undergone psychiatric or neurological evaluations. The patient held a college degree, studied abroad for two months, and worked part-time as a cashier during college. There was no history of abuse, trauma, or post-traumatic stress disorder, and the patient did not use alcohol, tobacco, or illegal drugs.

**Figure 1:**
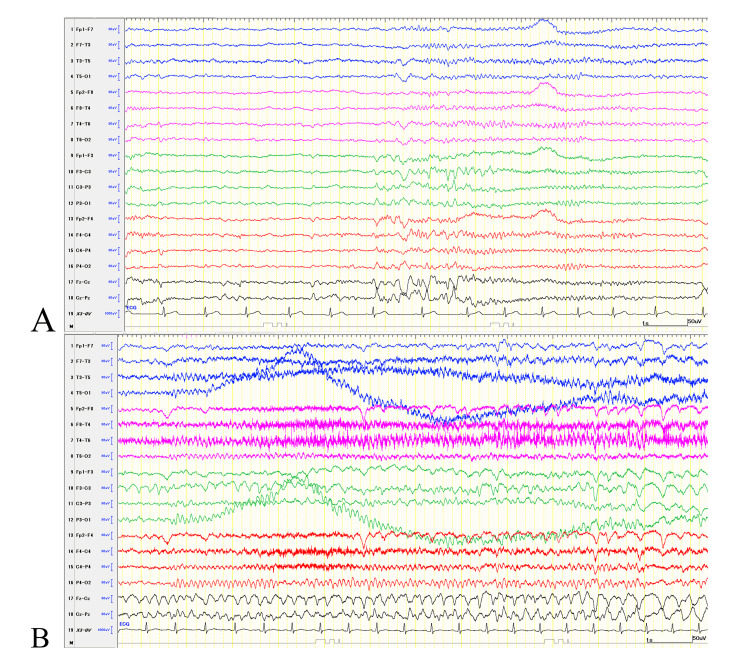
Interictal and ictal EEG. During wakefulness, EEG waveforms resembling vertex sharp transients were observed (A). At the onset of the ictal EEG, similar waveforms appeared in a repetitive pattern at C3 and Cz, corresponding to a left-handed clonic seizure. Twenty seconds after onset, the patient developed a focal to bilateral tonic-clonic seizure. The total duration was approximately 2 minutes (B). Display parameters: longitudinal bipolar montage, with a low-frequency filter of 0.53 Hz, a high-frequency filter of 120 Hz, and a page speed of 15 s. EEG: Electroencephalogram.

Physical and neurological examinations, blood tests, and brain MRI results were unremarkable. There were no findings suggestive of organic causes, including Parkinson's disease and stroke. The patient’s symptoms met the diagnostic criteria for apathy, characterized by a loss or decrease in motivation, decreased behavioral response to environmental stimulations, decreased curiosity about routine and novel events, and decreased emotional responses to positive and negative stimuli [9]. The patient did not show a depressed mood. A detailed mental status examination and psychological testing were difficult, as the patient was only able to respond to simple questions. Although the episodes of loss of consciousness that occurred later were suspected to be PNES, the initial symptoms of clonic seizure and tonic-clonic seizure were suspected to be epileptic seizures. After the discontinuation of medication starting on the admission day, VEEG monitoring was conducted from day 2 to day 5. VEEG revealed over ten focal clonic seizures and three focal to bilateral tonic-clonic seizures (Figure 1). After explaining a confirmed epilepsy diagnosis, LCM was reinstated at the same dosage on day 4. Later on the same day, the patient’s apathy improved significantly. The patient became able to speak spontaneously and independently perform daily activities such as eating and bathing. No epileptic seizures were recorded on VEEG after LCM resumed. The patient was discharged on day 9, after which psychiatric home nursing care was initiated.

After discharge, the patient showed gradual improvement in social activities, including shopping and cooking, and began communicating with her family, although she did not fully regain the pre-onset condition. Three months after discharge, the patient experienced their first post-discharge seizures, including focal clonic seizures and focal to bilateral tonic-clonic seizures. Thereafter, although her seizures occurred less than once a month, her social interactions and daily activities declined once again. Furthermore, the patient's mother resumed over-involvement. Additionally, although not triggered by specific situations and occurring on a monthly basis, the patient once again experienced episodes of loss of consciousness. The dosage of LCM was increased to 400 mg/day (200 mg twice daily); however, this adjustment was ineffective. Consequently, the patient required readmission four months after the prior discharge.

**Figure 2:**
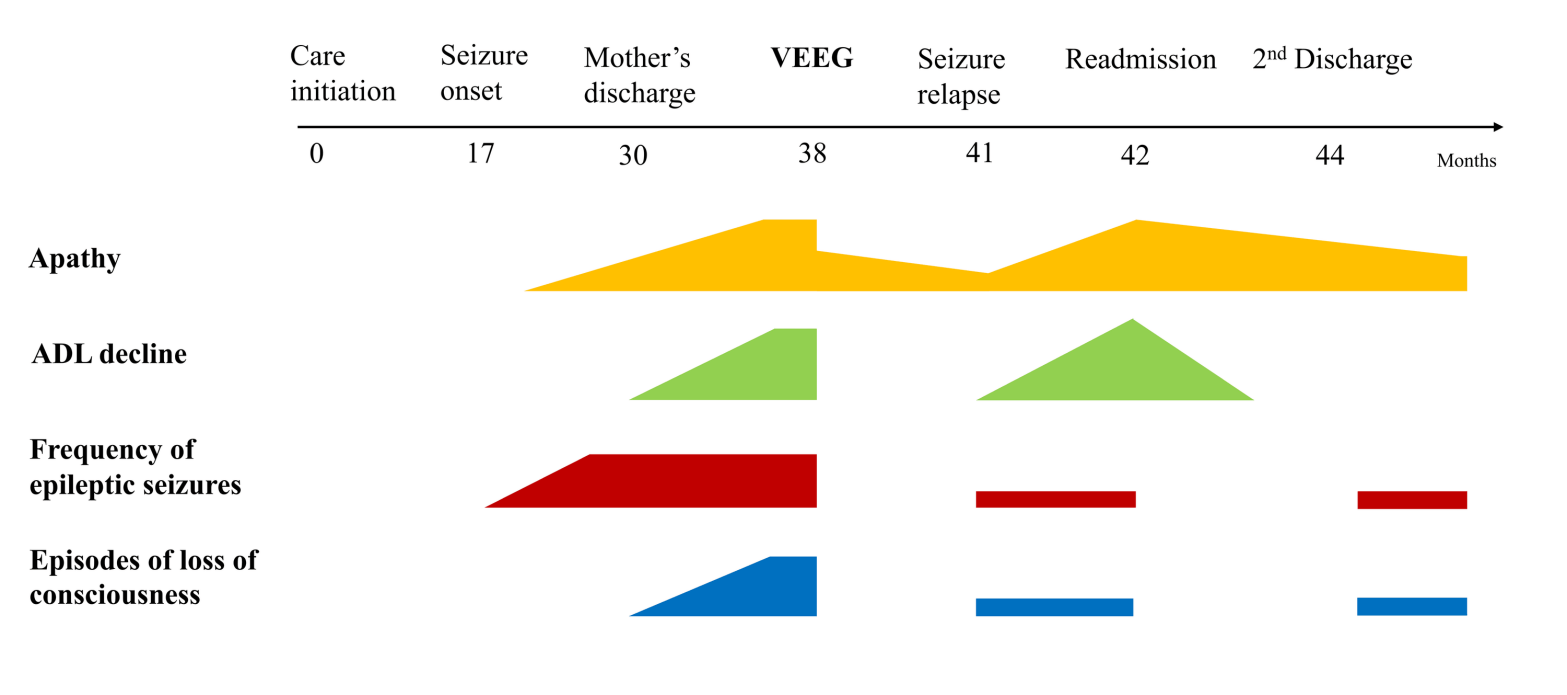
Chronological progression of the current medical history. ADL: Activities of daily living; VEEG: Video-electroencephalogram.

During hospitalization, LTG was introduced and titrated to 200 mg/day (100 mg twice daily) by day 23. On day 9 of hospitalization, VEEG was performed for four days; however, no seizures were recorded, nor were any seizures observed throughout the hospitalization. Occupational therapy (OT) and the promotion of independence helped the patient regain independence in daily activities, and by one month, the patient was nearly fully independent in daily life. Counseling was provided to the patient's mother, emphasizing the importance of avoiding over-involvement, as it could potentially hinder the patient's ability to maintain independence in daily activities. After two months of hospitalization, the patient was discharged with a plan to attend outpatient OT sessions on a weekly basis. One month post-discharge, the patient experienced focal clonic seizures as well as focal to bilateral tonic-clonic seizures, which have persisted on a monthly basis since then. Additionally, the episodes of loss of consciousness occurred on a monthly basis as well. However, there was no observed deterioration in the patient’s ADL. Figure 2 illustrates the chronological progression of the patient’s current medical history.

## Discussion

This case underscores the therapeutic potential of VEEG, which is known for its role in managing PNES and facilitating patients’ understanding of their condition, thereby addressing psychological conflicts. In people with PNES, the communication of a diagnosis should be clear, appropriate, and easily comprehensible to both patients and their families, emphasizing the importance of effective information delivery. In this context, providing explanations based on the results of VEEG represents a vital component [10]. Even for patients with epilepsy, providing explanations based on VEEG results could have a positive impact on psychiatric symptoms. Since psychological conflicts can lead to apathy [11], promoting a better understanding of epilepsy and correcting the misdiagnosis of seizures through VEEG may have alleviated the apathy. In this case, PNES was initially suspected based on the patient's medical history. However, PNES can coexist with epilepsy, and its presence does not rule out the possibility of epilepsy.

When apathy worsened again with seizures after discharge, VEEG was not effective. This outcome suggests that the initial effect of VEEG was primarily educational. We propose that the recurrence of seizures contributed to a decline in the patient’s self-esteem, which subsequently exacerbated apathy. The persistence of apathy, despite relatively good seizure control, combined with the observed effectiveness of psychiatric interventions such as OT and the encouragement of independence, indicates that apathy was strongly influenced by psychological factors. OT is considered effective in addressing apathy and reducing caregiver burden [12]. For this patient, attending OT outside the home was beneficial in preventing the re-exacerbation of apathy, as it helped reduce the mother's overinvolvement.

In the present case, the patient's apathy was severe. The patient’s family history of psychiatric disorders, combined with traits suggestive of ASD, likely predisposed her to psychological reactions to epilepsy [13,14]. These reactions may have been exacerbated by the mother’s excessive caregiving, which can contribute to anxiety and maladaptive behaviors in children [15]. Such overinvolvement often occurs in parents with mental health issues [16], making the mother's OCD a significant risk factor in this case. Furthermore, the patient’s characteristics associated with ASD may have intensified the mother’s OCD [14], reinforcing parental overinvolvement.

**Figure 3:**
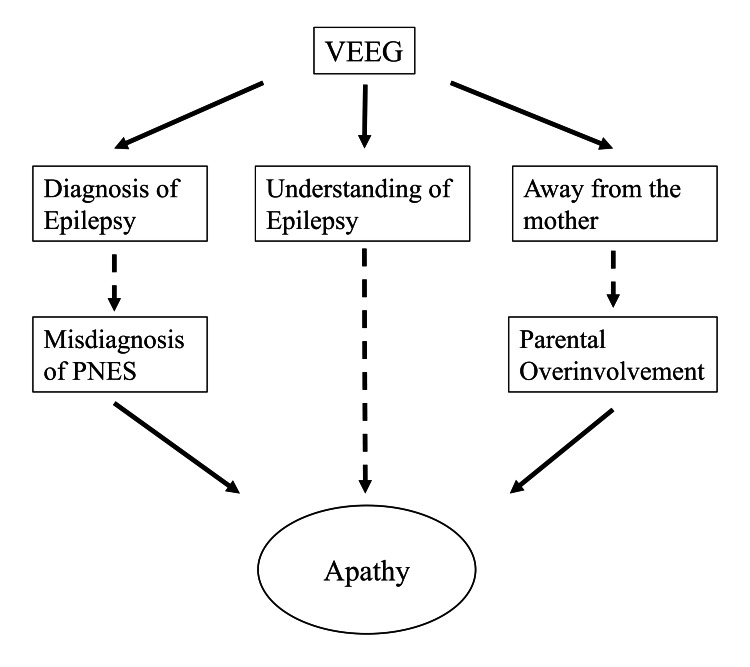
The therapeutic effects of diagnosis using VEEG and the causes of apathy in this case. Solid arrows denote enhancing effects, while dotted arrows represent reducing effects. VEEG: Video-electroencephalogram; PNES: Psychogenic nonepileptic seizures.

The frequency of epileptic seizures in the present case also decreased after VEEG, without any changes to antiepileptic medication. This might be explained by the fact that psychiatric disorders such as anxiety and depression correlate with the frequency of epileptic seizures [17]. The improvement in the patient’s apathy may have contributed to the reduction in seizure frequency. Therefore, the diagnosis made during the patient’s hospitalization, away from the mother's influence, may have enhanced the patient’s self-esteem, thereby potentially improving the patient’s apathy. Figure 3 shows the therapeutic effects of VEEG and the cause of apathy.

## Conclusions

This case emphasizes the critical role of accurate diagnosis in managing epilepsy and psychiatric comorbidities. VEEG not only facilitated a definitive diagnosis but also significantly improved the patient’s apathy and seizure control, highlighting its therapeutic impact. These findings suggest that VEEG may serve as an effective intervention for managing both psychiatric symptoms and epileptic seizures in complex cases. Further research is warranted to explore changes in psychiatric symptoms using standardized assessment scales before and after VEEG.
